# Spontaneously ruptured gastric gastrointestinal stromal tumor presenting as a large gastrohepatic mass

**DOI:** 10.11604/pamj.2026.53.69.49917

**Published:** 2026-02-09

**Authors:** Riya Yadav, Pratap Singh Parihar

**Affiliations:** 1 Department of Radiology, Datta Meghe Institute of Higher Education and Research (DMIHER), Wardha, India

**Keywords:** Ruptured hepatic mass, gastrohepatic region, hepatic lesion

Image in medicine

A 70-year-old female presented with complaints of abdominal pain and nausea for several days, without any known comorbidities or prior abdominal surgery. On examination, there was mild tenderness in the upper abdomen without palpable mass or hepatomegaly. Laboratory parameters, including liver function tests, were within normal limits. To further evaluate the cause of pain, contrast enhanced computed tomography (CECT) of the abdomen and pelvis was performed. Imaging revealed an ill-defined, multiloculated, heterogeneously enhancing lesion in the gastrohepatic region involving the left lobe of the liver (panel A, yellow arrow) measuring approximately 9.6x15.3x13.9cm. The lesion showed loss of fat planes with the adjacent stomach, pancreas and spleen with delayed peripheral enhancement (panel B, green arrow) and involvement of left branch of portal vein. Surrounding hepatic arterial hypertrophy was also noted. Associated findings included subdiaphragmatic air fluid collection (panel A, red arrow) and free intraperitoneal air (panel A, blue arrow), suggesting rupture of the lesion. Mild ascites and perihepatic lymphadenopathy were also noted. The imaging features, particularly the exophytic growth from the gastric wall, heterogeneous enhancement and contiguous hepatic involvement were suggestive of a ruptured gastrointestinal stromal tumor (GIST). Surgical resection was performed, and histopathological examination confirmed the diagnosis of GIST, showing spindle-shaped tumor cells positive for CD117 (c-KIT) and DOG1 immunomarkers. Gastrointestinal stromal tumors are the most common mesenchymal tumors of gastrointestinal tract, typically originating from the interstitial cells of Cajal and frequently associated with KIT or Platelet-Derived Growth Factor Receptor Alpha (PDGFRA) gene mutations. This case underscores the pivotal role of CECT in identifying complex gastrohepatic mass and their complications, guiding timely surgical management. Ruptured GISTs are rare but clinically significant due to the risk of peritoneal dissemination and recurrence. Early radiological diagnosis, prompt surgical management, and histopathological confirmation remain key to favorable clinical outcomes.

**Figure 1:**
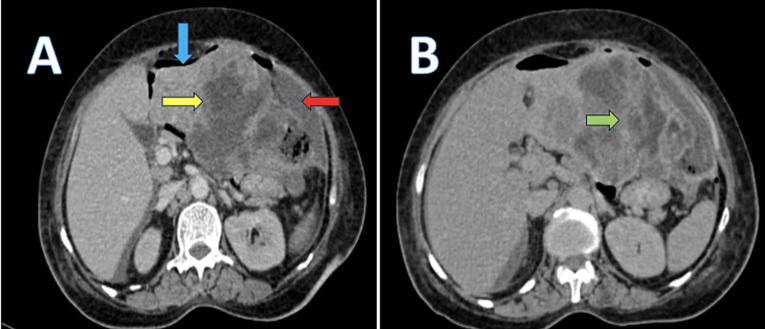
A) axial CECT upper abdomen venous phase showing ill-defined, multiloculated, heterogeneously enhancing lesion in the gastrohepatic region involving the left lobe of the liver (yellow arrow), subdiaphragmatic air fluid collection (red arrow), free intraperitoneal air (blue arrow); B) axial CECT upper abdomen delayed phase showing delayed phase enhancement (green arrow)

